# Preterm preeclampsia in relation to country of birth

**DOI:** 10.1038/jp.2016.73

**Published:** 2016-05-05

**Authors:** J G Ray, S Wanigaratne, A L Park, E Bartsch, S Dzakpasu, M L Urquia

**Affiliations:** 1 Keenan Research Centre, Li Ka Shing Knowledge Institute, St Michael's Hospital, Toronto, ON, Canada; 2 University of Toronto, Toronto, ON, Canada; 3 Institute for Clinical Evaluative Sciences, Toronto, ON, Canada; 4 Department of Obstetrics and Gynaecology, St Michael's Hospital, Toronto, ON, Canada; 5 Maternal and Infant Health Section, Public Health Agency of Canada, Ottawa, ON, Canada

## Abstract

**Objective:**

To examine the association between maternal country of birth and the risk of preeclampsia+preterm birth (PTB).

**Study Design:**

We completed a population-based study in the entire province of Ontario, where there is universal access to obstetrical care. We included 881 700 singleton livebirths among Canadian-born mothers and 305 547 births among immigrant mothers. Adjusted risk ratios (aRRs) were adjusted for maternal age, parity and income quintile.

**Results:**

Compared with a rate of preeclampsia+PTB of 4.0 per 1000 among Canadian-born mothers, the aRR of preeclampsia+PTB at 24 to 36 weeks was significantly higher for immigrant women from Nigeria (1.79, 95% confidence interval (CI) 1.12 to 2.84), the Philippines (1.54, 95% CI 1.30 to 1.86), Colombia (1.68, 95% CI 1.04 to 2.73), Jamaica (2.06, 95% CI 1.66 to 2.57) and Ghana (2.12, 95% CI 1.40 to 3.21). The aRRs generally followed a similar pattern for secondary outcomes. Specifically, women from Ghana were at highest risk of preeclampsia+very PTB (4.55, 95% CI 2.57 to 8.06), and women from Jamaica at the highest risk of preeclampsia+indicated PTB (1.89, 95% CI 1.43 to 2.50).

**Conclusion:**

The risk of preeclampsia+PTB is highest among women from a select number of countries. This information can enhance initiatives aimed at reducing the risk of PTB related to preeclampsia.

## Introduction

Preterm birth (PTB) heightens the risk of infant morbidity and mortality.^1, 2^ The causes of PTB may be divided into (1) spontaneous labor with intact membranes, (2) preterm premature rupture of membranes and (3) labor induction or Cesarean delivery for maternal or fetal indications (‘indicated', ‘provider-initiated' or ‘iatrogenic' preterm delivery).^1^ Approximately 25 to 30% of all PTBs are by indication, often in the presence of preeclampsia/eclampsia (henceforth ‘preeclampsia').^2, 3^ Specifically, ∼38% of indicated PTBs between 28 and 31 weeks are because of preeclampsia, as are 22% of indicated PTBs between 32 and 36 weeks.^4^ PTB infants are more likely to be growth restricted (adjusted odds ratio (OR) 2.3, 95% confidence interval (CI) 2.1 to 2.6), a risk that is much higher with indicated PTB (adjusted OR 6.4, 95% CI 5.5 to 7.5).^5^ Infants are much more likely be born small for gestational age in the presence of maternal preeclampsia and PTB at 33 to 36 weeks (OR 17.4, 95% CI 15.7 to 19.3), and especially when maternal preeclampsia and PTB occur before 33 weeks of gestation (OR 40.5, 95% CI 31.5 to 51.4), heightening the risk of infant mortality and morbidity^6^ and maternal morbidity.^7^ Thus, preeclampsia accompanied by PTB, especially indicated PTB, reflects a more pathological state with negative consequences for mother and child.

The risk of preeclampsia differs by maternal origin. We and others have observed a 2 to 3 times higher risk of preeclampsia among immigrant women from Caribbean and Sub-Saharan African world regions settling in Canada^8^ and other Western nations.^9, 10^ However, these studies mostly used broad categories of World regions of origin, rather than individual countries,^8, 9^ or they were conducted in settings without universal access to obstetrical care,^10^ and none assessed preeclampsia with PTB or PTB by indication.

Using data for the entire province of Ontario, Canada, where there is universal access to prenatal and obstetrical care, we examined the association between maternal country of birth and the risk of preeclampsia+PTB, including both very PTB and late PTB, as well preeclampsia+indicated PTB.

## Methods

### Study design

We completed a retrospective population-based cohort study, using unique encoded identifiers and analyzed at the Institute for Clinical Evaluative Sciences (ICES). ICES, an independent not-for-profit corporation, holds provincial health data on all of Ontario's citizens, including inpatient and outpatient services (see http://www.ices.on.ca/Data-and-Privacy/ICES-data/Types-of-ICES-Data). Ethics approval was granted by the research ethics board of Sunnybrook Health Sciences Centre, 30 January 2015, TRIM number 2012 0900 221 000.

### Participants

We included all singleton liveborn hospital-based births at 24 to 42 weeks of gestation in Ontario, from 1 April 2003 and 31 December 2012. As the birth was the unit of analysis, a woman may have contributed more than one birth in the study period. We excluded mothers aged <14 years or >50 years, nonresidents of Ontario at the time of delivery and women whose country of birth could not be determined. Infants weighing under 500 g at birth were also excluded, as were stillbirths and multi-fetal pregnancies.

### Exposures and outcomes

The main exposure of interest was maternal country of birth. The main study outcome was a diagnosis of preeclampsia+PTB at 24 to 36 completed weeks. Secondary outcomes included preeclampsia+late PTB at 34 to 36 weeks, preeclampsia+very PTB at 24 to 31 weeks as well as preeclampsia+indicated PTB at 24 to 36 weeks. Indicated PTB was identified as a PTB excluding preterm spontaneous labor with preterm delivery or premature rupture of membranes. We also evaluated the risk of preeclampsia between 24 and 42 weeks.

The diagnostic codes for preeclampsia (preeclampsia (O14) or eclampsia (O15)), as well as preterm labor (O601) or rupture of membranes (O42 and O756), were defined by the Canadian version of the International Statistical Classification of Diseases and Related Health Problems, 10th Revision (ICD-10-CA). These diagnostic codes are based on the presence of elevated blood pressure with proteinuria (preeclampsia) or new-onset seizure (eclampsia). In the time period of the current study, the definition of preeclampsia remained unchanged. Gestational age (in completed weeks) was recorded at the time of birth. Approximately 95% of Ontarian women undergo prenatal ultrasonography before 20 weeks of gestation, contributing to low misclassification of gestational age.^11^

### Database sources

All maternal and newborn infant hospitalizations were identified using the Canadian Institute for Health Information (CIHI) Discharge Abstract Database (DAD). The MOMBABY data set at ICES uses all DAD inpatient admission records of delivering mothers and their newborns between 2002 and 2013, and is described elsewhere.^8, 12, 13^ The MOMBABY data set contains the unique encrypted health-care number, maternal age, parity, up to 25 diagnoses coded by ICD-10-CA as well as gestational age at birth.

Women who delivered a liveborn singleton infant were linked (via encrypted health card number) to the Ontario portion of the federal Immigration, Refugees and Citizenship Canada (IRCC) Permanent Resident Database, also housed at ICES.^12, 13^ The IRCC Permanent Resident Database has records for permanent residents who immigrated to Ontario between 1985 and 2012. Although the father's country of birth is not known, we have shown a high concordance with the mother's World region of origin.^12^ Those births not linked to the IRCC Permanent Resident Database were identified as births to nonimmigrant women. More than 90% of this group comprises Canadian-born women, and henceforth is referred to as ‘Canadian-born'.

Residential postal code at the time of birth was used to determine the neighborhood income quintile, derived from Statistics Canada census data.

### Statistical analyses

The association between maternal country of birth and preeclampsia+PTB was analyzed using modified Poisson regression to generate risk ratios (RRs) and 95% CIs, comparing mother from each immigrant country with Canadian-born women (the referent). When modeling preeclampsia and preeclampsia+PTB (24 to 36 weeks), generalized estimating equations with an exchangeable correlation structure were used to account for the possible nonindependence of the outcome among women with more than one delivery (birth) in the data set. For the secondary outcomes, accounting for this nonindependence was not possible because of model nonconvergence, possibly because of lack of correlation in these outcomes among births to the same woman. RRs were adjusted (aRR) for maternal age (<20, 20 to 34, ⩾35 years), parity (0, 1, 2, 3, ⩾4, unknown) and residential income quintile (Q1 to Q5, unknown).

All statistical analyses were performed using SAS for UNIX, Version 9.2 (SAS Institute, Cary, NC, USA). The study was approved by the ethics review board of Sunnybrook Health Sciences Centre, Toronto, Ontario, Canada.

The study sample comprised all births in Ontario. No formal sample size calculations were required.

## Results

There were 881 700 singleton livebirths among 604 320 Canadian-born mothers and 305 547 births among 221 596 immigrant mothers (Table 1). Thus, 25.7% of all births were to immigrant women.

**Figure 1:**
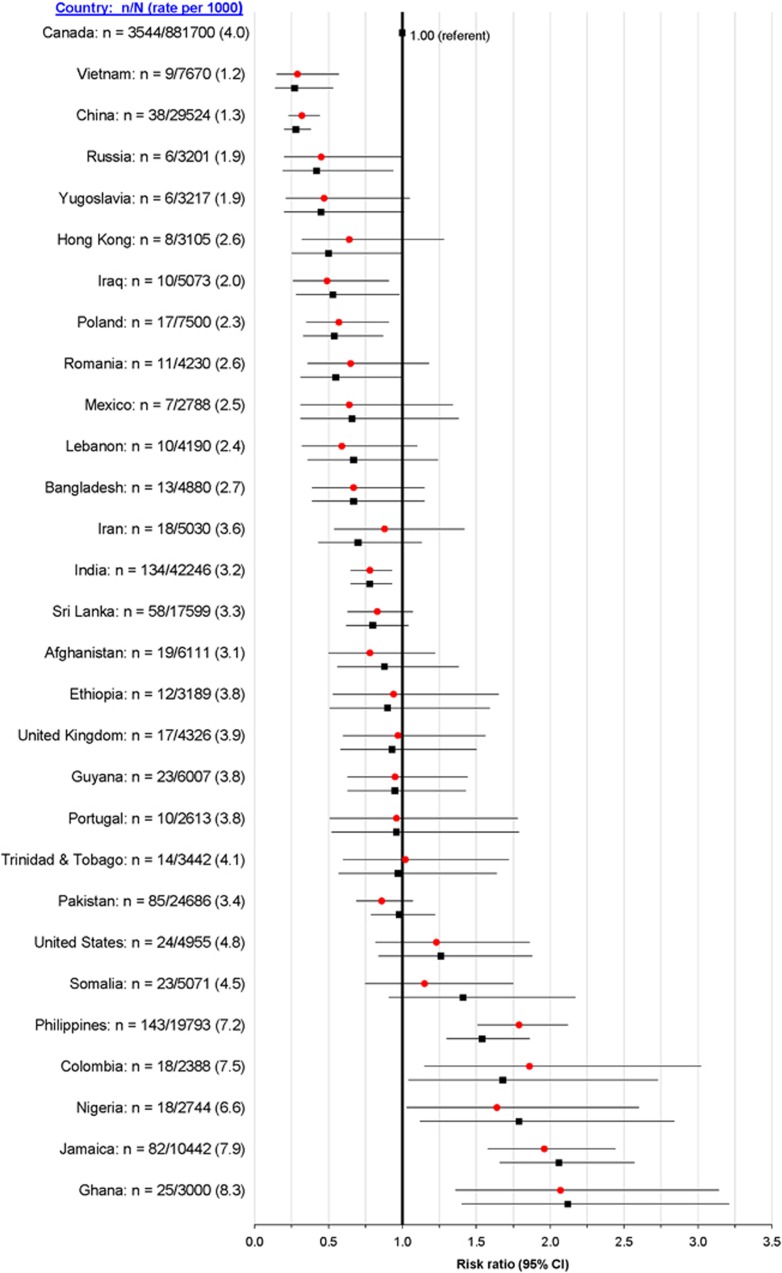
Rate and crude (red circles) and adjusted (black squares) risk ratios for preeclampsia+preterm birth at 24 to 36 weeks among all singleton livebirths. Risk ratios are adjusted for maternal age (<20, 20 to 34, ⩾35 years), parity (0, 1, 2, 3, ⩾4, unknown) and residential income quintile (Q1 to Q5, unknown).

Compared with births to Canadian-born mothers (4.0 per 1000), the rate of preeclampsia+PTB at 24 to 36 weeks was significantly higher for immigrant women from Nigeria (6.6 per 1000), the Philippines (7.2 per 1000), Colombia (7.5 per 1000), Jamaica (7.9 per 1000) and Ghana (8.3 per 1000) (Figure 1). The unadjusted and adjusted RRs generally followed this same pattern, with a doubling of the risk of preeclampsia+PTB among women from Jamaica and Ghana (Figure 1).

For the outcome of preeclampsia+late PTB at 34 to 36 weeks, women from the Philippines (aRR 1.34, 95% CI 1.06 to 1.70) and Jamaica (aRR 1.81, 95% CI 1.34 to 2.45) were at significantly elevated risk (Supplementary Figure S1), whereas for preeclampsia+very PTB at 24 to 32 weeks the aRRs remained higher for these two countries as well as Nigeria (aRR 2.58, 95% CI 1.15 to 5.76) and, especially, Ghana (aRR 4.55, 95% CI 2.57 to 8.06) (Supplementary Figure S2).

Compared with Canadian-born mothers (2.7 per 1000), preeclampsia+indicated PTB at 24 to 36 weeks was significantly more common in women from the Philippines (4.2 per 1000; aRR 1.34, 95% CI 1.07 to 1.67), Nigeria (4.4 per 1000; aRR 1.78, 95% CI 1.01 to 3.14) and Jamaica (4.8 per 1000; aRR 1.89, 95% CI 1.43 to 2.50) (Supplementary Figure S3).

The outcome of preeclampsia at 24 to 42 weeks followed a similar pattern to that above (Supplementary Figure S4).

## Discussion

### Main findings

In this population-based study of nearly 1.2 million singleton livebirths, the risk of preeclampsia+varying degrees of PTB was consistently highest in women from the Philippines, Nigeria and Jamaica. Women from Ghana had a 4.5 times higher risk of preeclampsia+PTB before 32 weeks, and women from Jamaica had nearly twice the risk of preeclampsia+indicated PTB.

### Strengths and limitations

The study outcomes of preeclampsia and timing of delivery were accurately captured in the current administrative databases, as shown by Canadian validation study, with a sensitivity of 75% and a specificity of 99% for preeclampsia.^14^ Similarly, in a large Danish validation study of a hospital data set akin to ours, the sensitivity, specificity and positive predictive value for preeclampsia was 69, 99 and 74%, respectively.^15^ In the decade of our study, the definition of preeclampsia remained unchanged, and 99% of women in Ontario received antenatal care and delivered in a hospital. Thus, preeclampsia may have been under-ascertained herein, but when identified, women were correctly labeled with the condition. Preeclampsia was just as likely to be diagnosed in immigrant and Canadian-born mothers, as all women were enrolled in universal health care.

In our study, the ethnicity of Canadian-born mothers was not known; yet, most were born to British and European parents, the predominant ethnic group in Canada 25 to 35 years earlier.^16^ This is supported by our observed rate of preeclampsia+PTB of 4.0 per 1000 among Canadian-born mothers, nearly identical to the rate of 3.9 per 1000 in women from the United Kingdom (Figure 1). Notwithstanding the point above, the two largest non-white ethnic groups among Canadian-born women are Chinese and South Asians. By including women of Chinese or Indian ethnicity in our Canadian-born reference group, our aRRs would be shifted closer to 1.0. As the Canadian-born group contains a small proportion of immigrants who arrived in Ontario before 1985, largely comprising those from European countries,^17^ misclassification within the comparison group should not bias our results. Our study was limited to singleton livebirths, whereas multi-fetal pregnancies or those ending in stillbirth may have different outcomes, in terms of preeclampsia risk or timing of delivery.^18^ Moreover, we did not report on neonatal outcomes for the PTBs.

Within the province of Ontario, in which 1 in 4 births are to immigrants,^12, 13^ we had a rare opportunity to link immigrant status to other health databases. For countries that contribute a large number of immigrants to our population, we were able to systematically and precisely evaluate the relation between maternal country of origin and preeclampsia+PTB risk. For very PTB or indicated PTB, fewer countries could be included.

### Mechanisms

Chronic inflammatory and placental vaso-occlusive lesions are more commonly seen on placental pathology with preeclampsia+PTB than with spontaneous PTB.^19^ These differences reflect divergent mechanisms not only for the type of PTB—the former largely indicated PTB and the latter spontaneous PTB—but also for the impact on fetal and newborn health.^1, 5, 6, 7^ The elevated risk of preeclampsia+PTB, especially indicated PTB, observed herein among specific immigrant (or country of birth) groups may reflect a higher susceptibility to a placental disorder. Certainly, a direct assessment of placental pathology could shed light about why certain immigrant groups (for example, Chinese women) are at lower risk, and others from the same World region (for example, Filipina women) are at much higher risk of preeclampsia+PTB. Although we did not adjust for duration of residence among immigrants, large differences would not be expected across countries, as most immigrant women to Canada give birth within 10 to 12 years after arrival.^8^

We intentionally chose to not control for maternal obesity or chronic hypertension. Although they may differ by country of birth, each is in the causal pathway between maternal country of birth and risk of preeclampsia, rather than behaving as true confounders. For example, women from the Philippines have higher rates of chronic hypertension and obesity than women from China,^20^ and each of these conditions predisposes to preeclampsia.^21^ Certainly, unexplored anthropometric measures, dietary and smoking practices may partly explain the difference in the risk of preeclampsia+PTB between countries of origin, as might the heritability of preeclampsia that has been estimated to be as high as 50%.^22^

### Interpretation

Women from the Ghana, the Philippines, Nigeria and Jamaica are at higher risk of preeclampsia+very PTB, and the latter three countries are also at higher risk of preeclampsia+indicated PTB. Our findings complement those of others who have studied the relation between maternal World region of birth and the risk of preeclampsia,^8, 9, 10^ but our results also highlight the need to concentrate on each particular country of origin. For example, there is a marked difference in the risk of preeclampsia between women from the Philippines vs those from China (Supplementary Figure S4), and the magnitude of this difference is more pronounced when preeclampsia is accompanied by indicated PTB (Supplementary Figure S3). Similarly, women from Somalia—that, although geographically part of Sub-Saharan Africa, is also part of the Arab world—were at no higher risk of preeclampsia+PTB, whereas those from the more Western countries of Ghana and Nigeria, were.

These findings should not alter the general provision of prenatal care to all women, including blood pressure measurements at each visit. However, ongoing research is needed to determine whether a woman's country of origin can enhance how we screen for^23, 24^ and attempt to prevent preeclampsia—especially preterm onset of preeclampsia.^25^ Although we identified some immigrant groups at higher risk of preeclampsia in Ontario, other nationalities may be associated with a higher risk in other high immigration settings. Country of origin may be used as a flag to further inquire about individual risk factors to refine clinical decision making, particularly among recent immigrant women who may not have a complete obstetric history.

## Figures and Tables

**Table 1:** Characteristics of singleton livebirths at 24 to 42 weeks of gestation in Ontario, 2003 to 2012

*Characteristic*	*Births to Canadian-born mothers (*n=*881* *700 births)*	*Births to immigrant mothers (*n=*305* *547 births)*
Number of women who delivered	604 320	221 596
		
*At the index delivery*		
* *Mean (s.d.) age, years	29.7 (5.6)	30.6 (5.2)
* Age category, years*		
<25	162 979 (18.5)	37 706 (12.3)
25 to 34	542 500 (61.5)	195 771 (64.1)
⩾35	176 221 (20.0)	72 070 (23.6)
* Parity*		
0	403 120 (45.7)	128 308 (41.2)
1	312 687 (35.5)	114 074 (37.3)
2	114 114 (12.9)	42 474 (13.9)
3	33 526 (3.8)	13 246 (4.3)
⩾4	18 057 (2.1)	7392 (2.4)
* *Unknown	196 (0.0)	53 (0.0)
* Residential income quintile (Q)*		
Q1 (lowest)	162 875 (18.5)	101 061 (33.1)
Q2	166 886 (18.9)	70 622 (23.1)
Q3	183 042 (20.1)	58 639 (19.2)
Q4	197 220 (22.4)	47 523 (15.6)
Q5 (highest)	166 876 (18.9)	27 212 (8.9)
Unknown	4801 (0.5)	492 (0.2)
		
*Of the newborn infant*		
* *Female sex	429 292 (48.7)	148 086 (48.5)
Mean (s.d.) gestational age at birth, weeks	38.9 (1.7)	38.8 (1.7)

All data are presented as number (%) unless otherwise indicated.
