# Supplementary material for: Preterm preeclampsia in relation to country of birth
Source: J Perinatol. 2016 May 5;36(9):718–22. doi: 10.1038/jp.2016.73 (PMC5007604; doi:10.1038/jp.2016.73)

Figure S1. Rate and adjusted risk ratios for preeclampsia + late preterm birth at 34-36 weeks among all singleton livebirths. Risk ratios are adjusted for maternal age (< 20, 20-34, ≥ 35 years), parity (0, 1, 2, 3, ≥ 4, unknown) and residential income quintile (Q1-Q5, unknown). Data were suppressed if there were < 6 outcome events.

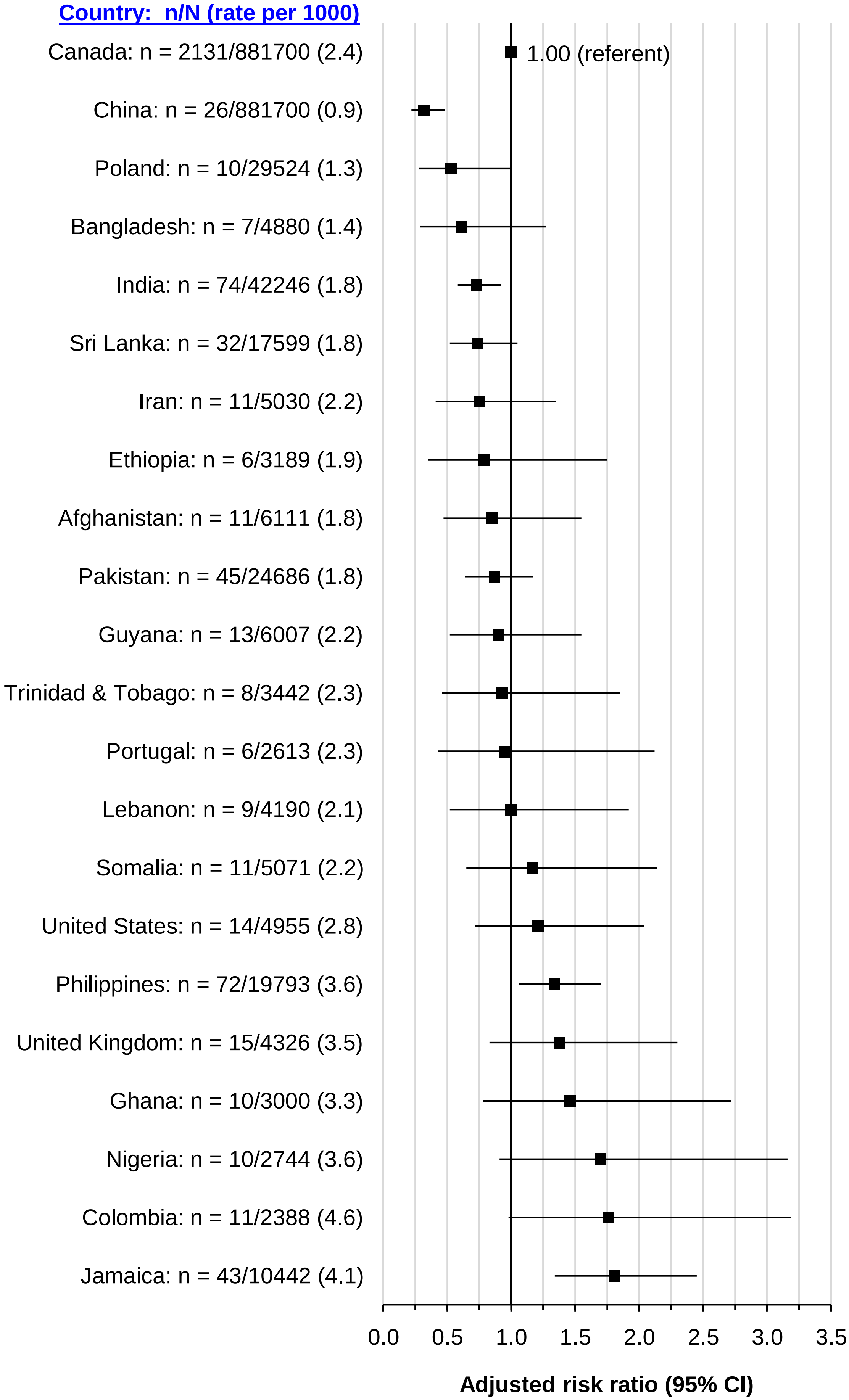

Supplement: Supplementary Figure S1 [file jp201673x1.pdf]
